# Cooperative roles of the suprachiasmatic nucleus central clock and the adrenal clock in controlling circadian glucocorticoid rhythm

**DOI:** 10.1038/srep46404

**Published:** 2017-04-12

**Authors:** Sooyoung Chung, Eun Jeong Lee, Hyo Kyeong Cha, Jeongah Kim, Doyeon Kim, Gi Hoon Son, Kyungjin Kim

**Affiliations:** 1 Department of Brain and Cognitive Sciences, Scranton College, Ewha Womans University, Seoul, Korea; 2 Department of Brain and Cognitive Sciences, Daegu-Gyeongbuk Institute of Science & Technology (DGIST), Daegu, Korea; 3 Korea Brain Research Institute (KBRI), Daegu, Korea; 4 Department of Biomedical Sciences, College of Medicine, Korea University, Seoul, Korea

## Abstract

The mammalian circadian timing system consists of the central clock in the hypothalamic suprachiasmatic nucleus (SCN) and subsidiary peripheral clocks in other tissues. Glucocorticoids (GCs) are adrenal steroid hormones with widespread physiological effects that undergo daily oscillations. We previously demonstrated that the adrenal peripheral clock plays a pivotal role in circadian GC rhythm by driving cyclic GC biosynthesis. Here, we show that the daily rhythm in circulating GC levels is controlled by bimodal actions of central and adrenal clockwork. When mice were subjected to daytime restricted feeding to uncouple central and peripheral rhythms, adrenal GC contents and steroidogenic acute regulatory protein expression peaked around zeitgeber time 00 (ZT00), consistent with shifted adrenal clock gene expression. However, restricted feeding produced two distinct peaks in plasma GC levels: one related to adrenal GC content and the other around ZT12, which required an intact SCN. Light pulse-evoked activation of the SCN increased circulating GC levels in both wild-type and adrenal clock-disrupted mutant mice without marked induction of GC biosynthesis. In conclusion, we demonstrate that adrenal clock-dependent steroidogenesis and a SCN-driven central mechanism regulating GC release cooperate to produce daily circulatory GC rhythm.

Most physiological and behavioral events in mammals exhibit daily oscillations generated by an internal time-keeping system composed of clock genes with interacting positive and negative feedback loops. The hypothalamic suprachiasmatic nucleus (SCN) harbors a master clock that synchronizes and maintains the circadian rhythms of the periphery^1^,^2^. Glucocorticoids (GCs; primarily corticosterone in rodents and cortisol in primates), which are secreted by adrenocortical steroidogenic cells in a rhythmic fashion, have widespread effects on the body including gluconeogenesis, lipid metabolism, cardiovascular tone, inflammation, and immune functions. GCs mediate behavioral adaptations to external cues by responding to stress via activation of the hypothalamic-pituitary-adrenal (HPA) axis and following a circadian rhythm^3^,^4^,^5^. In the circadian timing system, GCs function as a key humoral mediator that transmits resetting signals from the SCN to peripheral clocks. Therefore, circulating GC levels are tightly regulated by the SCN and directly affect the molecular clockwork in target tissues^6^,^7^,^8^.

As abrogation of the SCN completely eliminates daily rhythms in plasma adrenocorticotropic hormone (ACTH) and GCs, the oscillatory patterns of GCs in the periphery have been primarily attributed to the SCN^9^. Notably, SCN graft transplantation into SCN-lesioned hamsters restores circadian rhythmicity of locomotion but not of GC release, implying that synaptic connectivity may be required for SCN control of adrenal rhythms^10^. The SCN may also directly transmit photic information to the adrenal gland. For instance, Okamura and colleagues showed that diurnal changes in GC levels require splanchnic nerve integrity but are not mediated by differential responsiveness to ACTH^11^.

On the other hand, several lines of evidence strongly support the presence of adrenal-intrinsic mechanisms involving the adrenal peripheral clock. Oster and colleagues proposed the existence of a gating mechanism in the adrenal gland based on the circadian nature of its sensitivity to upstream regulators^12^,^13^. It is also noteworthy that analyses of adrenal gene expression clearly define the presence of a canonical molecular clock in adrenocortical cells and further show oscillatory patterns in the expression of sets of genes related to GC production and secretion^14^,^15^. Our previous study demonstrates that the transcription of steroidogenic acute regulatory protein (StAR), a rate-limiting factor of adrenal steroid biosynthesis, follows a circadian rhythm similar to that of circulating GCs under the control of a CLOCK:ARNTL heterodimer in adrenocortical cells. Selective abrogation of the adrenal clock flattens the rhythm in StAR expression and decreases adrenal GC production, which attenuates the rhythm of GCs in plasma^16^.

Considering these complicated features, multimodal mechanisms may underlie circadian GC rhythm, primarily involving actions of the SCN through both neural and endocrine systems as well as adrenal-intrinsic mechanisms. However, the dissection of the roles of each component has been difficult due to hierarchical interactions between the SCN central pacemaker and the adrenal peripheral clock. In the present study, we examined the functional consequences of disentangling the SCN master clock and adrenal peripheral clock, thereby demonstrating how multiple mechanisms integrate to produce a robust GC rhythm in circulation.

## Results

### Altered locomotor behavior and adrenal clock gene expression with daytime restricted feeding

**Figure 1:**
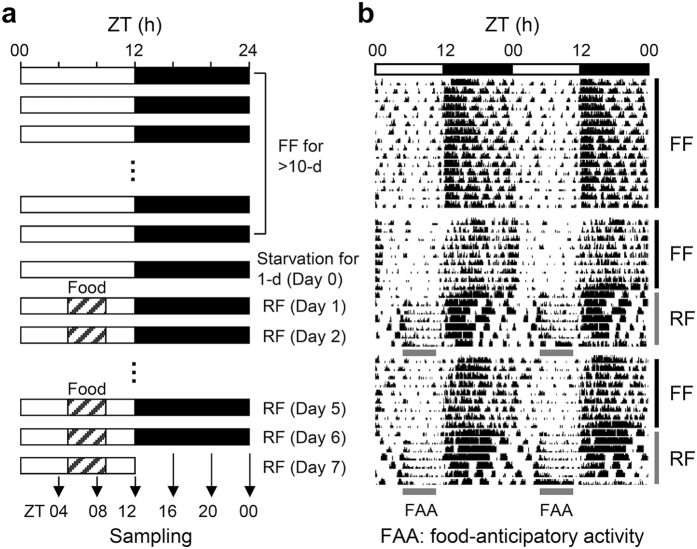
Daytime restricted feeding regimen. (**a**) Schematic depiction of the restricted feeding regimen. After starvation for 1 day, mice were fed exclusively during the day (ZT05 to 09) for 7 consecutive days. During the last day, mice were sacrificed at 4-hour intervals. (**b**) Locomotor activities of freely fed (FF) and restricted fed (RF) mice.

Feeding nocturnal rodents exclusively during the day disrupts synchrony between the SCN master pacemaker and peripheral oscillators. This daytime restricted feeding inverts the phases of clock gene expression cycles in peripheral organs such as the liver, kidney, heart, and pancreas but barely alters those in the SCN^17^. Therefore, we employed daytime restricted feeding to uncouple the phases of peripheral oscillators from the SCN pacemaker. Mice were fed during 4 hours in the daytime (zeitgeber time (ZT)05 to 09) under normal light/dark conditions (Fig. 1a). In contrast to freely fed mice, mice on restricted feeding for more than 3 consecutive days showed increased spontaneous locomotor activity during the daytime, known as food-anticipatory activity, indicating food-dependent entrainment (Fig. 1b).

**Figure 2:**
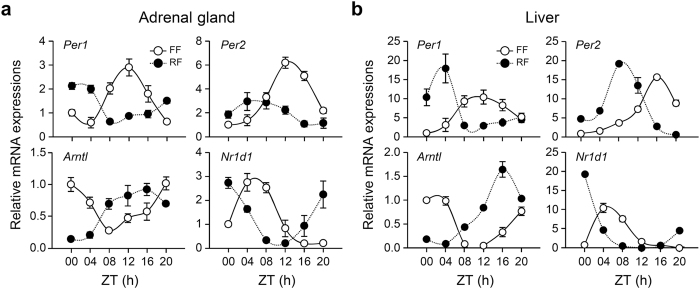
Effect of restricted feeding on clock gene expression in the adrenal gland and liver. Daily profiles of *Per1, Per2, Arntl,* and *Nr1d1* mRNA expression in the adrenal gland (**a**) and liver (**b**) of freely fed (FF) and restricted fed (RF) mice were determined by quantitative RT-PCR. Data were normalized to levels of TBP and expressed as mean ± SE (n = 4).

We next examined whether daytime restricted feeding influences cyclic gene expression in the adrenal gland. Mice subjected to restricted feeding for 6 consecutive days were sacrificed at 4-hour intervals, and cyclic mRNA expression of canonical clock genes was examined in the adrenal gland and liver. The phases of cyclic mRNA species including *Per1, Per2, Arntl,* and *Nr1d1* were shifted by 4 to 8 hours in both the adrenal gland (Fig. 2a) and liver (Fig. 2b) compared with those in freely fed mice. These results demonstrate that food functions as a strong zeitgeber for the adrenal peripheral clock and can disentangle adrenal oscillators from the central rhythm produced by the SCN.

### Effect of daytime restricted feeding on adrenal StAR expression and GC rhythm

**Figure 3:**
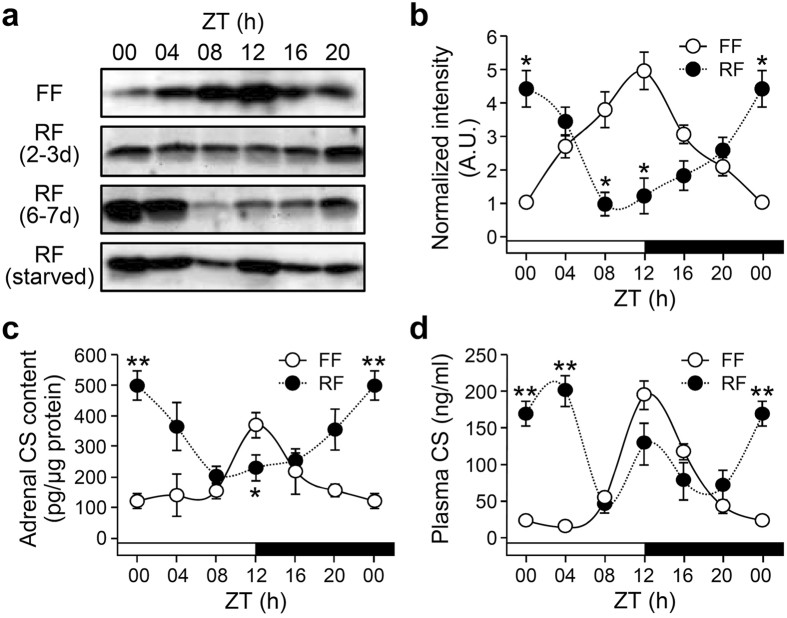
Effect of restricted feeding on adrenal StAR expression and corticosterone levels. (**a** and **b**) Adrenal StAR protein levels in freely fed (FF) and restricted fed (RF) mice as determined by immunoblotting. Mice subjected to restricted feeding were sacrificed on day 3 (2–3 d) or day 7 (6–7 d). Restricted fed (starved) mice were sacrificed on day 7 without food on the last day to examine the possible effect of hunger on StAR expression (**a**). Optical band intensities for FF and RF mice (6–7 d) are expressed as mean ± SE of arbitrary units (A.U.), with values from FF mice at ZT00 defined as 1 (**b**; n = 3; *p < 0.05 *vs.* FF at the same time-of-day). (**c** and **d**) Effects of restricted feeding on daily rhythm of adrenal corticosterone content (**c**; n = 7–10; *p < 0.05 and **p < 0.01 *vs.* FF at the same time-of-day) and circulating corticosterone levels in plasma (**d**; n = 4–5; **p < 0.01 *vs.* FF at the same time-of-day) as measured by radioimmunoassay.

We previously demonstrated that StAR gene transcription and accompanying adrenal corticosterone contents correlate with circulating corticosterone levels in an adrenal clock-dependent fashion in freely fed mice^16^. As phases of adrenal clock gene expression shift after daytime restricted feeding for more than 6 days, we examined whether the same duration of restricted feeding also affects diurnal StAR expression and corticosterone rhythms. Restricted feeding gradually shifted diurnal StAR protein expression (Fig. 3a and b), with 6 days being sufficient to invert the rhythms (Fig. 3a) and adrenal corticosterone content (Fig. 3c) compared with those in freely fed mice. The increased StAR expression around ZT00 was unlikely due to hunger-related stress, as plasma ACTH levels were not significantly altered by feeding regimen (Supplementary Fig. S1) and, more importantly, starvation on day 7 produced a substantial increase in StAR protein expression at ZT12 (Fig. 3a, bottom panel). However, restricted feeding produced split circulatory corticosterone rhythms, with two distinct peaks at ZT00 and 12 (Fig. 3d).

**Figure 4:**
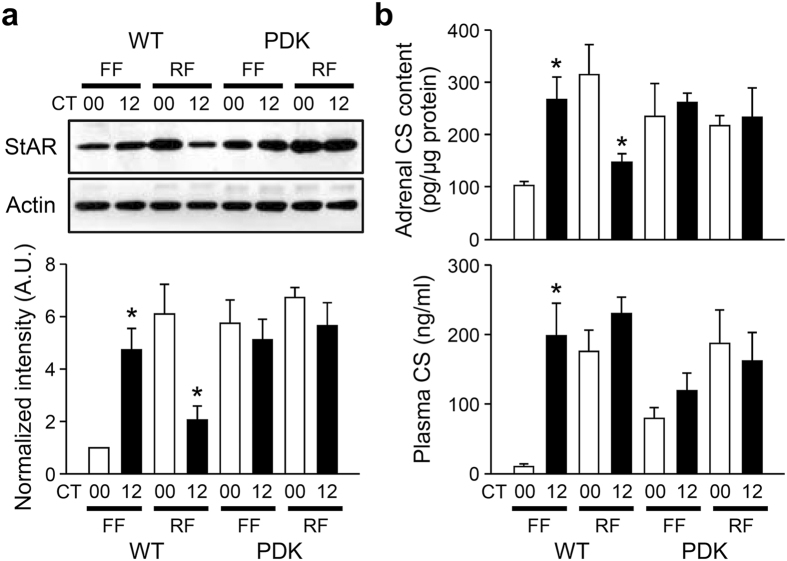
Effect of restricted feeding on adrenal rhythms of PDK mice. (**a**) Representative images of adrenal StAR protein levels at CT00 and 12 in freely fed (FF) and restricted fed (RF) mice as determined by immunoblotting (upper). Optical band intensities are expressed as mean ± SE of arbitrary units (A.U.), with values from WT mice at CT00 defined as 1 (lower; n = 3; *p < 0.05 *vs.* CT00 of the same feeding regimen). (**b**) Adrenal corticosterone contents (upper; n = 4) and circulating corticosterone levels (lower; n = 4–6) in WT and PDK mice (*p < 0.05 *vs.* CT00 of the same genotype and feeding regimen).

The restricted feeding-induced dissociation between corticosterone profiles in the circulation and adrenal lysates suggests that multiple regulatory mechanisms underlie the GC rhythm. It is noteworthy that daytime restricted feeding-induced food-anticipatory activity did not depend on either the SCN central pacemaker or functional clock genes^18^,^19^. Considering that StAR links adrenal GC biosynthesis with adrenal molecular clock as demonstrated in our previous study^16^, we thus examined the effect of daytime restricted feeding on StAR expression as well as daily variations in corticosterone profiles in clock-defective *Per1*^−/−^; *Per2*^−/−^ (Per double knockout, PDK) mutant mice. When we compared subjective morning (circadian time (CT)00) and evening (CT12) levels, freely fed PDK mice exhibited no diurnal variations in adrenal StAR expression (Fig. 4a and also see Supplementary Fig. S2a for 4-point per day profiles), adrenal corticosterone contents (Fig. 4b, top panel), or circulating corticosterone levels (Fig. 4b, bottom panel and Supplementary Fig. S2b), and restricted feeding did not affect the arrhythmic features of adrenal functions in mutant mice. These results suggest that daytime feeding-induced alterations in adrenal corticosterone production require functional circadian clock machinery and adrenal StAR expression and that subsequent steroidogenesis may contribute to restricted feeding-induced high levels of circulating corticosterone at ZT00.

### Role of the SCN in daytime restricted feeding-induced adrenal StAR expression and GC rhythm

**Figure 5:**
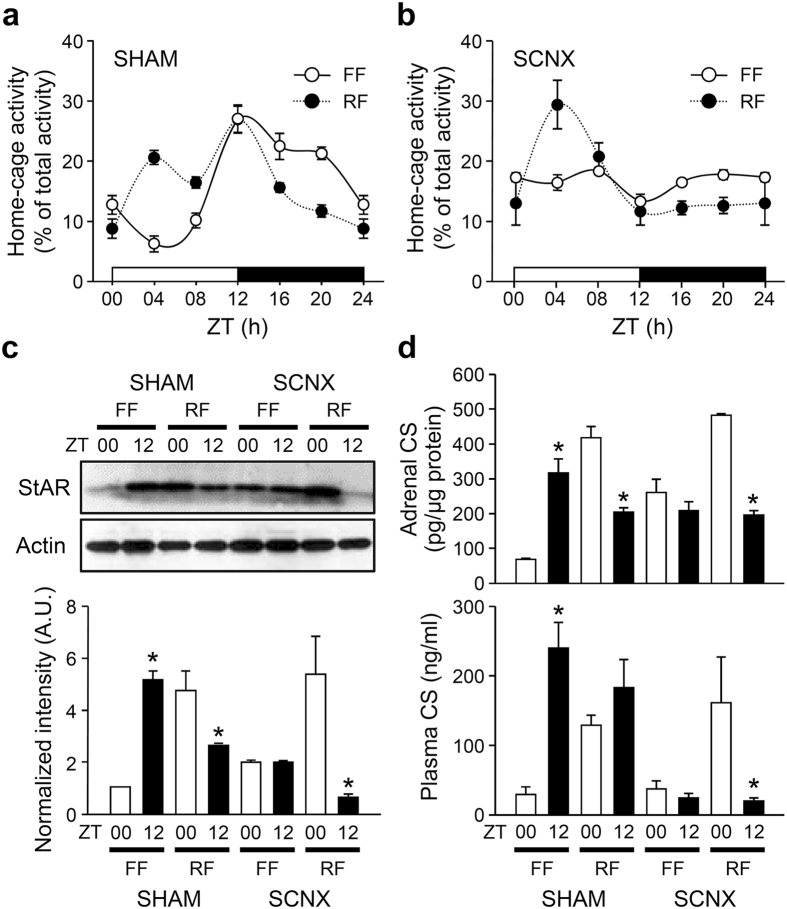
Effect of restricted feeding on behavioral and adrenal rhythms of SCNX mice. (**a** and **b**) Home cage activity of freely fed (FF) and restricted fed (RF) sham-operated (SHAM; **a**) or SCNX (**b**) mice (n = 6). (**c**) Representative images of adrenal StAR protein levels at ZT00 and 12 (upper). Optical band intensities are expressed as mean ± SE of arbitrary units (A.U.), with values from WT mice at ZT00 defined as 1 (lower; n = 4; *p < 0.05 *vs.* ZT00 of the same treatment). (**d**) Adrenal corticosterone contents (upper; n = 4) and circulating corticosterone levels (lower; n = 4–6) in SHAM and SCNX mice (*p < 0.05 *vs.* ZT00 of the same treatment).

The SCN plays a key role in light-dependent entrainment of the circadian timing system and is barely influenced by feeding schedule^17^. Therefore, we tested whether the SCN central clock is responsible for the split pattern of corticosterone secretion induced by daytime feeding. The locomotor activity of freely fed sham-operated mice followed an apparent diurnal rhythm, and restricted feeding induced food-anticipatory activity (Fig. 5a). These behavioral rhythms disappeared in SCN-lesioned (SCNX) mice, even under a normal light-dark photoperiod (Fig. 5b). However, the restricted feeding of SCNX mice evoked locomotor rhythms entrained to the feeding schedule, with higher activity during the light period and lower activity during the dark period. Restricted fed SCNX mice thus exhibited normal food-anticipatory activity between ZT00 and 04, which agrees with a previous report^18^. StAR protein expression (Fig. 5c and Supplementary Fig. S2c) and corticosterone contents in the adrenal gland (Fig. 5d, top panel) were not significantly different between ZT00 and ZT12 in freely fed SCNX mice, with intermediate levels between the nadir and peak of freely fed SHAM mice. However, restricted feeding induced significant StAR protein expression and adrenal corticosterone content in the adrenal gland of the SCNX mice at ZT00. Furthermore, the mismatch between adrenal corticosterone contents and circulating corticosterone levels disappeared in the daily fed SCNX mice, with plasma corticosterone profiles similar to those of StAR expression and adrenal corticosterone contents (Fig. 5d and Supplementary Fig. S2d). Together, these findings suggest that the SCN contributes to the peak levels of circulating corticosterone at ZT12 regardless of feeding regimen.

**Figure 6:**
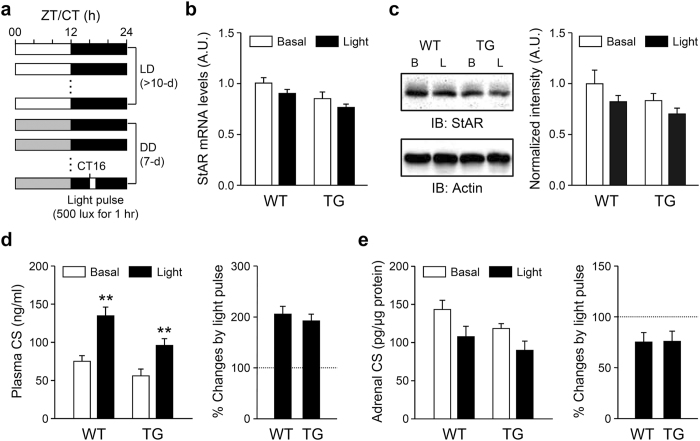
Increased plasma corticosterone levels without marked induction of adrenal steroidogenesis by a light pulse. (**a**) Schematic depiction of the light pulse experimental protocol. (**b** and **c**) Adrenal StAR mRNA (**b**; n = 5–9) and protein (**c**; n = 4) expression levels induced by photic stimulation. (**d**) Similar increases in plasma corticosterone levels by light stimuli in WT and TG mice (n = 12–15; **p < 0.01 *vs.* basal levels of the same genotype). (**e**) Adrenal corticosterone contents in response to the photic stimulus (n = 7–10).

We then examined whether the SCN directly regulates corticosterone secretion in the absence of substantial steroidogenesis and the adrenal peripheral clock. Okamura and colleagues showed that photic activation of the SCN central clock during early subjective nighttime (CT16) induces *Per1* expression in the adrenal gland and increases circulating GC levels^11^. Notably, they also reported that the photic signal induces *Nr5a1* gene transcript encoding steroidogenic factor-1, a transcriptional activator of various steroidogenic genes including StAR^11^. We employed a similar photic stimulation scheme (Fig. 6a) in adrenal clock-disrupted transgenic (TG) mice, in which *Arntl* expression is selectively attenuated in the adrenal gland^16^ compared with wild-type (WT) littermates. A light pulse for 1 hour did not induce adrenal StAR mRNA (Fig. 6b) or protein (Fig. 6c) levels but significantly increased plasma corticosterone levels by approximately 2-fold in both WT and TG mice (Fig. 6d). By contrast, adrenal corticosterone contents only showed a tendency to be reduced by the light pulse in both WT and TG mice (Fig. 6e). It is therefore plausible that activation of the SCN by a light pulse may evoke corticosterone secretion from the adrenal gland without steroidogenesis via an adrenal clock-independent mechanism.

## Discussion

The present study demonstrates that diurnal GC production and secretion from the adrenal gland is under the bimodal control of the SCN pacemaker and an adrenal-intrinsic oscillator. Restricted feeding during the daytime, which uncouples central and peripheral rhythms^17^, inverted the daily rhythm of plasma GC levels, implying that distinct regulatory mechanisms are cooperatively involved. Whereas the early peak in corticosterone levels was closely linked to changes in adrenal steroidogenesis, the later peak was independent of rhythmic GC production but required an intact SCN. Furthermore, photic activation of the SCN increased plasma GC levels without requiring either an increase in adrenal steroidogenesis or a functional molecular clock in adrenocortical GC-producing cells.

Multiple regulatory mechanisms are believed to underlie adrenal rhythms, primarily due to the relatively restricted roles of upstream hormonal regulators of the HPA axis^12^,^20^. Whereas pulsatile and stress-induced GC release are mainly regulated through the HPA axis by a negative feedback loop to upstream hormonal regulators, SCN-controlled autonomic innervation and adrenocortical circadian clocks are required for daily GC rhythms. For instance, splanchnic nerve transection reduces the peak of plasma GC rhythm^12^,^21^. The SCN-derived autonomic nervous system also mediates photic stimulation of the adrenal gland, thereby resetting the adrenal local clock and rapidly increasing plasma GC levels^11^. In addition to this neural mechanism, the adrenal-intrinsic oscillator directs the circadian rise of circulating GC levels by regulating diverse nodes of steroidogenesis and intracellular signaling cascades that mediate periodic GC production and controls a gating mechanism involved in sensitivity to ACTH^13^,^16^. However, it is not yet fully understood how SCN-driven signaling and adrenal-intrinsic mechanisms coordinate GC production and release to produce robust GC rhythm.

Daytime restricted feeding promotes food-anticipatory activity and uncouples local clocks in peripheral tissues and extra-SCN brain regions from the central SCN pacemaker in nocturnal rodents^17^,^22^,^23^. Consistent with previous reports, we found that 6 days of daytime restricted feeding was sufficient to cause food-anticipatory activity and shift clock gene expression in the liver. The phases of clock gene expression in the adrenal gland were also entrained by restricted access to food, consistent with a previous finding by Spencer and colleagues^24^. Notably, the adrenal gland has been regarded as a ‘second-order pacemaker’ that is strongly influenced by autonomic signals from the SCN central pacemaker and that controls multiple peripheral oscillators through steroid signaling^25^. The phases of oscillators in such organs including the pituitary, pineal, and submaxillary salivary glands are less affected by the food-entrainable oscillator that mediates food-anticipatory activity and shifts clock gene expression in other peripheral tissues, and more affected by sympathetic signals from the SCN^18^,^26^. Although the autonomic nervous system is proposed to play an important role in the synchronization of adrenal rhythm^25^, certain metabolic and/or endocrine cues exerted by daytime restricted feeding may also influence the phase of the adrenal oscillator.

Along with clock gene expression in the adrenal gland, diurnal alterations in adrenal GC content and periodic expression of StAR protein were also shifted by daytime feeding. The nature of the diurnal rhythm in adrenal steroidogenic gene expression is controversial. For example, several reports, including our previous study, demonstrate rhythmic StAR expression in rodent models^16^,^24^,^27^,^28^,^29^, whereas some microarray data do not show a rhythmicity of adrenal StAR transcription^13^,^14^,^30^. This discrepancy may be due to the relatively modest rhythmicity in adrenal StAR expression compared with that of canonical clock genes. Nevertheless, it should be noted several independent studies have proposed the transcriptional control of StAR expression by the CLOCK:ARNTL heterodimer^15^,^16^,^31^. Altered steroidogenesis in association with adrenal StAR expression likely influences circulating GC levels, as an increment in plasma corticosterone levels was observed around ZT00 (a morning peak), when adrenal corticosterone content reached its peak. However, the second peak at ZT12 (an evening peak), when circulating GC levels were high in freely fed mice, was still present in mice subjected to daytime restricted feeding, despite its reduced magnitude. These findings agree with our previous report, in which suppressed ARNTL expression in adrenocortical GC-producing cells was found to flatten rhythmicity in adrenal StAR expression and corticosterone content but only attenuate plasma corticosterone rhythm^16^.

Alterations in GC production and release by daytime restricted feeding evidently require an intact circadian clockwork, as food-induced rhythms were not found in arrhythmic PDK mice. However, restricted feeding-induced daily rhythms in locomotor activity, adrenal StAR expression, and corticosterone content were found regardless of whether the SCN was intact; the evening peak in plasma GC levels disappeared in SCN-lesioned mice, whereas the morning peak in corticosterone content remained. More importantly, photic activation of the SCN central clock revealed the presence of a steroidogenesis-independent and SCN-driven mechanism stimulating acute GC release from the adrenal gland. A previous study shows that a light pulse during the early night activates the SCN and subsequently evokes an HPA axis-independent increment in plasma GC levels^11^. We found that a photic stimulus acutely increases plasma corticosterone levels by approximately 2-fold even in TG mice that exhibit reduced adrenal ARNTL expression^16^. Nevertheless, adrenal StAR expression and corticosterone content were not significantly changed in either genotype. There was even a tendency toward reduced adrenal corticosterone contents in both groups, suggesting that corticosterone release was evoked by photic activation of the SCN without steroidogenesis. These findings collectively suggest that an activating signal from the SCN involving enhanced GC secretion contributes to a circadian rise in circulating GC levels at the beginning of the active period regardless of the phase of adrenal rhythms.

The SCN-driven signal appears to activate the adrenal gland to release GCs, most plausibly by a neural mechanism involving the sympathetic nervous system, but it is still unclear how this neural signal acutely regulates adrenal GC secretion. Medullary-cortical signaling pathways via catecholamines, neuropeptides, and intra-adrenal blood flow have been proposed as mediators of sympathetic nerve-dependent adrenal activation^32^. Also, an intact adrenal medulla, which is a primary target of preganglionic sympathetic fibers, may be required for proper circadian peaks of plasma GCs^12^. Considering that decreased corticosterone content was associated with photic signal-evoked corticosterone secretion, such neural mechanisms may activate the adrenal gland to acutely release corticosterone from the stored pool. It has long been thought that steroid hormone secretion is primarily controlled by the regulation of steroidogenesis through concentration gradient-dependent diffusion. Several previous studies, however, point to an intracellular retention of steroids against a concentration gradient at intracellular sites proximal to the plasma membrane and propose a possible steroid transport mechanism involving organic anion transport^33^,^34^. More recently, Rettori and colleagues suggested that GCs could be acutely released from a storage location after exposure to stress, and this release may be mediated by the paracrine actions of prostaglandins and subsequent production of nitric oxide^35^. The researchers’ ultrastructural analysis revealed a larger number of microcytotic vesicles and filopodia-like structures of the cell membrane in adrenal glands exposed to stress. Also, invaginations in close contact with mitochondria, lipid droplets, and additional microcytotic vesicles frequently formed in activated adrenocortical cells. Therefore, it is plausible that medullary-cortical interactions and accompanying intracellular signals leading to the rapid release of stored steroids may underlie the SCN-driven and steroidogenesis-independent increase in circulating GC levels.

In conclusion, we demonstrated bimodal regulatory mechanisms of circadian circulatory GC rhythms by disentangling the roles of the SCN central pacemaker in timely control of steroid secretion and the adrenal peripheral oscillator related to periodic steroidogenesis. In this regard, it is of interest to note that the cellular mechanisms underlying circadian GC rhythm are similar to those found in insulin secretion. The cell autonomous clock in pancreatic cells plays a key role in the robust daily rhythms of insulin secretion by coordinating the periodic expression of gene transcripts involved in the assembly, trafficking, and membrane fusion of vesicles required for secretion of peptide hormones^36^, indicating differential secretory mechanisms between steroid and peptide hormones as well as cell type-specific roles of local clockworks. Therefore, the circadian rhythms of GC biosynthesis and secretion are tightly controlled by multiple regulatory mechanisms. Considering the functional significance of GC rhythms in orchestrating physiology, behavior, and the circadian timing system in mammals, our findings suggest that circadian rhythms and their complex control mechanisms should be considered when attempting to understand physiological and pathophysiological conditions linked to changes in basal GC secretion.

## Methods

### Animal care and handling

Male C57BL/6 J mice at 9–12 weeks of age were mainly used in this study. WT or mutant mice^16^ were kept in temperature-controlled (22–23 °C) quarters under a 12-h light/dark photoperiod (lights on at 8:00 a.m.) with standard mouse chow and water available *ad libitum*. Spontaneous home-cage activity was monitored using a VitalView^®^ data acquisition system (Mini Mitter, Bend, OR) with implantable E-Mitters. Daytime restricted feeding was carried out as previously described with modifications^17^. Mice were fed exclusively during the day from ZT/CT05 to 09. For some mice, a bilateral thermal lesion of the SCN was performed stereotaxically as previously described^11^. For light exposure experiments, we applied incandescent light (500 lux, 1 hour beginning at ZT16) to conscious, freely moving mice, which were maintained in constant dark for 7 days after entrainment for more than 10 days under normal light/dark conditions. Mice were sacrificed within 30 min of an indicated time-of-day except ZT00; to avoid possible alarm responses to light-on, we sacrificed animals between ZT23 and 00 for this time point. Use of animals and related experimental procedures were approved by the Institutional Animal Care and Use Committee of Korea University (KU-IACUC-20130219-2). All experiments were performed in accordance with guidelines and regulations of the KU-IACUC.

### Hormone measurement

Corticosterone levels in adrenal lysates and plasma samples were assayed using a commercial corticosterone radioimmunoassay kit (DPC, Los Angeles, CA) as previously described^37^.

### RNA isolation and reverse transcription-polymerase chain reaction (RT-PCR)

RNA analysis was performed as previously described with modifications^16^,^38^. Mouse tissue was rapidly removed and frozen in liquid nitrogen. Total RNA was isolated by the single-step acid guanidinium thiocyanate-phenol-chloroform method, and 500 ng of each RNA sample was reverse-transcribed with MMLV reverse transcriptase (Promega, Madison, WI). Aliquots of cDNA were subjected to quantitative RT-PCR in the presence of SYBR Green I (Sigma, St. Louis, MO). Gene expression levels were normalized to those of TATA box-binding protein (TBP). Primer sequences used for real-time RT-PCR were as follows: *Star* up, 5′-TTG GGC ATA CTC AAC AAC CA-3′; *Star* dn, 5′-GAA ACA CCT TGC CCA CAT CT-3′; *Per1* up, 5′-GTG TCG TGA TTA AAT TAG TCA G-3′; *Per1* dn, 5′-ACC ACT CAT GTC TGG GCC-3′; *Per2* up: 5′-ATG CTC GCC ATC CAC AAG A-3′; *Per2* dn: 5′-GCG GAA TCG AAT GGG AGA AT-3′; *Arntl* up, 5′-CAA GCA CCT TCC TTC CAA TG-3′; *Arntl* dn, 5′-GAT TGC AGT CCA CAC CAC TG-3′; *Nr1d1* up: 5′-AGG GCA CAA GCA ACA TTA CC-3′; *Nr1d1* dn: 5′-CAC AGG CGT GCA CTC CAT AG-3; *Tbp* up: 5′-GGG AGA ATC ATG GAC CAG AA-3′; *Tbp* dn: 5′-CCG TAA GGC ATC ATT GGA CT-3′.

### Immunoblot analyses

Anti-StAR (Abcam, Cambridge, UK) and actin (Sigma) antibodies were commercially available. Whole-cell extracts were resolved on sodium dodecyl sulfate-polyacrylamide gels and transferred to polyvinylidene fluoride membranes (Millipore, Bedford, MA). The blots were blocked in Tris-buffered saline (10 mM Tris, pH 7.6, 150 mM NaCl, and 2 mM MgCl_2_) containing 0.3% Tween 20 and 3% bovine serum albumin and incubated with primary antibody at room temperature for 1 hour. Antibody binding was detected by incubation with secondary antibodies linked to horseradish peroxidase (Jackson ImmunoResearch Laboratories, West Grove, PA) accompanied by visualization using enhanced chemiluminescence reagents (Thermo Fisher Scientific, Waltham, MA). Optical densities of immunoreactive bands were quantified using NIH ImageJ software (downloaded from http://rsb.info.nih.gov/ij/), and the relative amounts of target proteins were deduced by comparison with optical band densities from serially diluted reference extracts.

### Statistical analysis

StAR protein expression, plasma corticosterone levels, and adrenal corticosterone contents were statistically evaluated with Student’s *t*-tests. Significance was set at p < 0.05.

## Additional Information

**How to cite this article**: Chung, S. *et al*. Cooperative roles of the suprachiasmatic nucleus central clock and the adrenal clock in controlling circadian glucocorticoid rhythm. *Sci. Rep.* **7**, 46404; doi: 10.1038/srep46404 (2017).

**Publisher's note:** Springer Nature remains neutral with regard to jurisdictional claims in published maps and institutional affiliations.

## Supplementary Material

Supplementary Information
