# Supplementary material for: Cooperative roles of the suprachiasmatic nucleus central clock and the adrenal clock in controlling circadian glucocorticoid rhythm
Source: Sci Rep. 2017 Apr 12;7:46404. doi: 10.1038/srep46404 (PMC5388859; doi:10.1038/srep46404)
Supplement: Supplementary Information [file srep46404-s1.pdf]

Supplementary figures for SREP-16-36990A:

Cooperative roles of the suprachiasmatic nucleus central clock and the adrenal clock in controlling circadian glucocorticoid rhythm

Sooyoung Chung[1], Eun Jeong Lee[2,3], Hyo Kyeong Cha[4], Jeongah Kim[2], Doyeon Kim[2], Gi Hoon Son[4,*], Kyungjin Kim[2,3,*]

[1]Department of Brain and Cognitive Sciences, Scranton College, Ewha Womans University, Seoul, Korea.

[2]Department of Brain and Cognitive Sciences, Daegu-Gyeongbuk Institute of Science & Technology (DGIST), Daegu, Korea

[3]Korea Brain Research Institute (KBRI), Daegu, Korea

[4]Department of Biomedical Sciences, College of Medicine, Korea University, Seoul, Korea

Supplementary Figure S1. Chung *et al.*

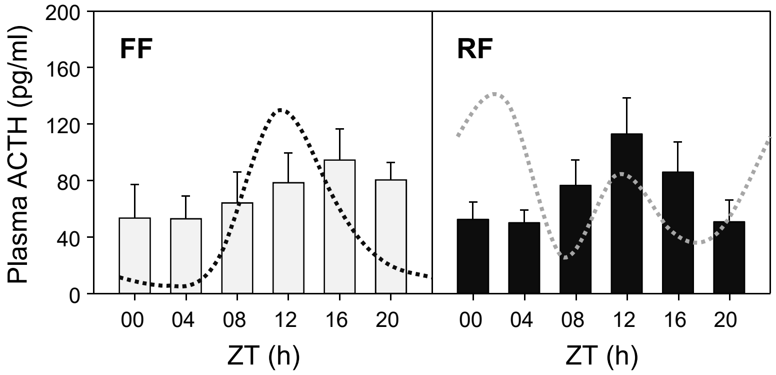

Figure S1. Effect of restricted feeding on plasma ACTH levels. Plasma ACTH levels in freely fed (FF) and restricted fed (RF for 6-7 days) mice as determined using a commercial ELISA kit (MD Bioproducts, Oakdale, MN) are expressed as mean ± SE (n=7-10). Dashed lines indicate corticosterone profiles shown in Figure 3d.

Supplementary Figure S2. Chung *et al.*

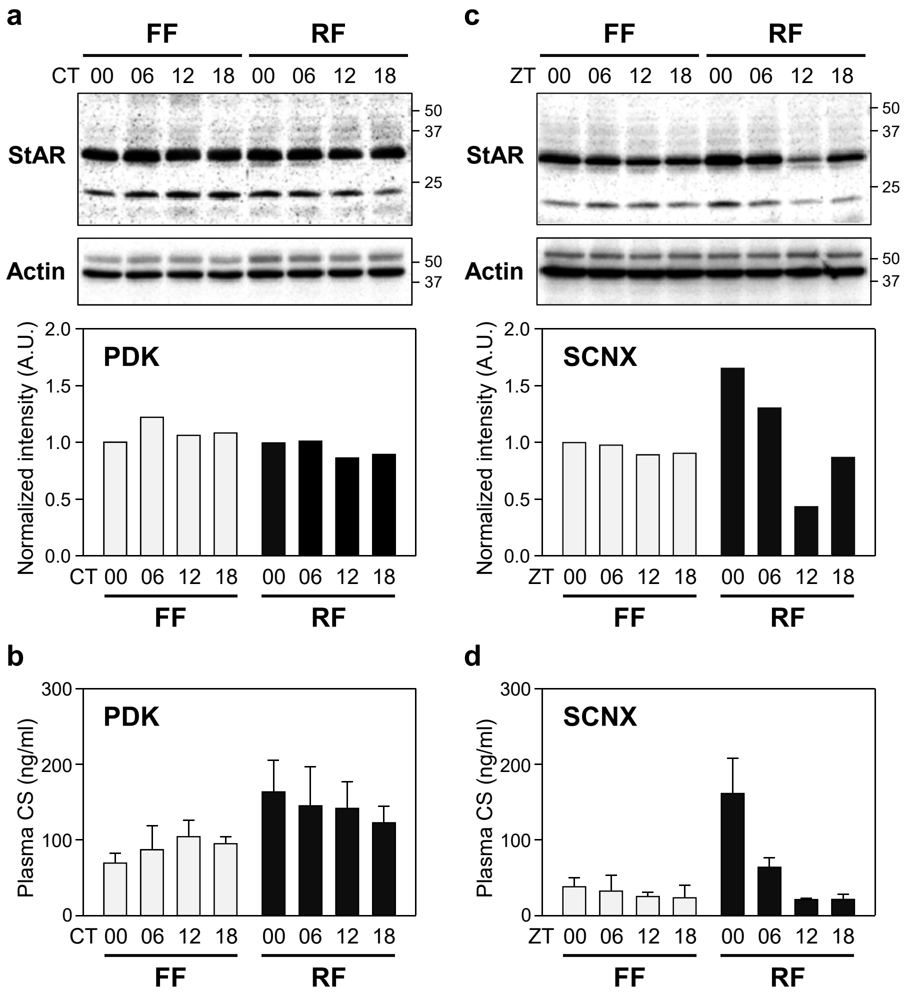

Figure S2. Effects of restricted feeding on adrenal rhythms of PDK mice and SCNX mice. (a) Immunoblot analysis of adrenal StAR protein levels at CT00, 06, 12, and 18 in freely fed (FF) and restricted fed (RF) PDK mice. Optical band intensities are expressed as mean ± SE of arbitrary units (A.U.), with values from FF mice at CT00 defined as 1 in the lower panel. We pooled and homogenized two adrenal glands from different animals for each point. (b) Plasma corticosterone levels in FF and RF PDK mice (n=4-5 for CT00 and 12, and n=2 for CT06 and 18). (c) Immunoblot analysis of adrenal StAR protein levels at ZT00, 06, 12, and 18 in SCNX mice subjected to FF or RF. Optical band intensities are expressed as mean ± SE of A.U., with values from FF mice at ZT00 defined as 1 in the lower panel. We pooled and lysed two adrenal glands from different animals for each point. (d) Plasma corticosterone levels in FF and RF PDK mice (n=4 for ZT00 and 12, and n=2 for ZT06 and 18). Corticosterone levels at CT/ZT00 and 12 are the same as those presented in Figures 4b (for PDK mice) and 5d (for SCNX mice).

Supplementary Figure S3. Chung *et al.*

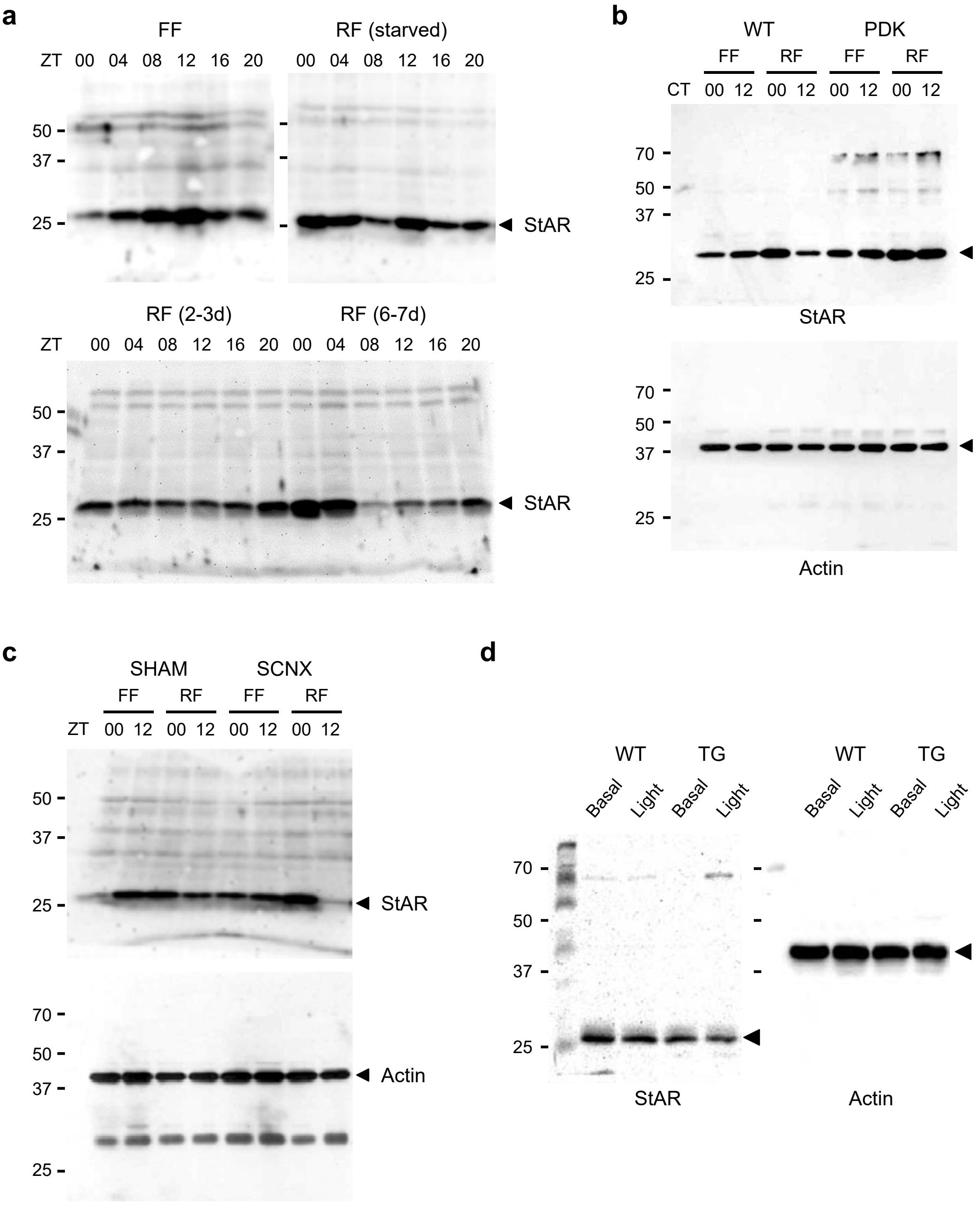

Figure S3. Full-length blots for immunoblot analyses. Full-length blots for Figures 3a (a), 4a (b), 5c (c), and 6c (d). The estimated molecular weights are marked on the left side of each blot. Arrowheads indicate the immunoreactive bands for target proteins of interest.
